# Research on Key Algorithms of the Lung CAD System Based on Cascade Feature and Hybrid Swarm Intelligence Optimization for MKL-SVM

**DOI:** 10.1155/2021/5491017

**Published:** 2021-09-03

**Authors:** Jiayue Chang, Yang Li, Hewei Zheng

**Affiliations:** School of Computer Science and Engineering, Changchun University of Technology, Jilin 130012, China

## Abstract

Feature selection and lung nodule recognition are the core modules of the lung computer-aided detection (Lung CAD) system. To improve the performance of the Lung CAD system, algorithmic research is carried out for the above two parts, respectively. First, in view of the poor interpretability of deep features and the incomplete expression of clinically defined handcrafted features, a feature cascade method is proposed to obtain richer feature information of nodules as the final input of the classifier. Second, to better map the global characteristics of samples, the multiple kernel learning support vector machine (MKL-SVM) algorithm with a linear convex combination of polynomial kernel and sigmoid kernel is proposed. Furthermore, this paper applied the methods for speed contraction factor and roulette strategy, and a mixture of simulated annealing (SA) and particle swarm optimization (PSO) is used for global optimization, so as to solve the problem that the PSO is easy to lose particle diversity and fall into the local optimal solution as well as improve the model's training speed. Therefore, the MKL-SVM algorithm is presented in this paper, which is based on swarm intelligence optimization is proposed for lung nodule recognition. Finally, the algorithm construction experiments are conducted on the cooperative hospital dataset and compared with 8 advanced algorithms on the public dataset LUNA16. The experimental results show that the proposed algorithms can improve the accuracy of lung nodule recognition and reduce the missed detection of nodules.

## 1. Introduction

Lung cancer deaths account for 25% of all cancer deaths worldwide [1]. With regard to the number of lung cancer patients, China ranks first in the world, accounting for 37% of the total global cases [2]. The latest research data show that in China, both male and female lung cancer mortality rates rank first, male morbidity ranks first, and female morbidity ranks second [3]. The 5-year relative survival rate of lung cancer patients is 18%, and early surgery is the most effective treatment for lung cancer. If health management can be strengthened to achieve early screening, detection, and early treatment of lung cancer, the cure rate of patients can reach 65%, which can effectively improve the survival rate of lung cancer patients and avoid missing the best treatment opportunity [4].

Computed tomography (CT) is an important method for detecting early lung cancer. On CT images, early lung cancer appears as a round or round-like dense shadow with a diameter of less than 30 mm, known as a lung nodule. The Lung CAD system is a comprehensive application of medical image processing and machine-learning technology, aiming to detect nodules and identify benign and malignant lung nodules from CT quickly and accurately, so as to provide efficient assisted diagnosis and treatment solutions. The standard Lung CAD system usually includes image preprocessing, lung parenchymal segmentation, segmentation of a candidate nodule region of interest (ROI) or volume of interest (VOI), the calculation and selection of ROI or VOI features, and benign or malignant pulmonary nodule recognition.

Many machine-learning algorithms are applied to Lung CAD systems, which are mainly divided into two categories: one is the traditional machine-learning algorithms, such as random forest (RF), support vector machine (SVM), k-nearest neighbor (KNN) algorithm; the other one is deep learning algorithms, among which convolutional neural networks (CNN) is the most widely used, such as VGG16 [5], U-Net [6], and ResNet [7]. Traditional machine-learning methods generally design texture features, morphological features, and other handcrafted features according to doctors' suggestions, and then input them into the appropriate classifier, of which SVM is the most commonly used [8–11]. SVM is a traditional machine-learning method, mainly applied to small sample data, with strong interpretability and deep theoretical foundation [12]. The complexity of SVM computation only depends on the number of support vectors, not the dimensionality of the space, which avoids the disaster of dimensionality to a certain extent. Kernel function is the necessary theoretical tool of SVM, which can map original data to high-dimensional feature space and realize nonlinear transformation. Compared with single kernel learning, MKL-SVM can improve classification accuracy and robustness [13]. However, SVM is difficult to implement for large-scale data and is very sensitive to the setting of kernel function parameters. With the great success of deep learning in medical image processing, classification based on the CNN method to automatically learn features is very attractive [14]. However, a deep learning system trained from scratch requires a lot of training data, and it is still a challenge to obtain such large medical images with detailed annotations [15]. The deep model training phase requires longer training time and more complex processing, as well as higher requirements for the selection of computing devices, so it is mostly implemented through transfer learning [16]. The theoretical basis of deep learning is still not perfect and lacks interpretability, but it is widely used due to its good results. Although the above related algorithms have achieved certain results in Lung CAD recognition, there are still the following challenges:

1. Deep features extracted by deep convolutional neural networks can improve the detection effect, but the training process requires a large amount of data, and the design of the model is very complicated and computationally expensive. Although transfer learning can solve the time-consuming problem of training the model from scratch, it still lacks some interpretability for data features.
2. The traditional SVM algorithm realizes the nonlinear mapping of high-dimensional space through the kernel function, so designing the suitable kernel function for a specific problem is still difficult.
3. The performance of SVM is strongly influenced by the parameters, so finding the suitable parameters can improve the performance of the SVM and avoid falling into the local optimum. In general, the most common global optimization algorithm for SVM is the grid search method, which tries every possibility through loop traversal to select the best performing parameter set, but the disadvantage is that the calculation is large and the search time is long. Swarm intelligence optimization algorithms provide a solution to this problem, of which the PSO algorithm is very extensive. However, PSO is a local search algorithm; as the number of iterations increases, it is easy to lose the diversity of particles and fall into the local optimal solution.

Both traditional machine-learning methods and deep learning methods have their own characteristics, so specific solutions need to be proposed for specific problems. In recent years, traditional machine-learning algorithms are often used in combination with deep learning methods; for instance, SVM is often used as a classifier for the hybrid architecture of deep learning models, and has achieved remarkable results [17–20]. In addition, algorithms are still limited in the field of medical image analysis; it is worth exploring them in combination with classical techniques in other fields. For example, in recent years, attention mechanisms commonly used in the field of text parsing [21], embedded real-time detection strategy in the field of quality monitoring [22], siamese networks in video tracking [23], graph neural networks in knowledge graphs [24], all of them provide valuable references for the subsequent wise information technology of medicine.

In this study, the key algorithms for feature extraction and lung nodule recognition in the Lung CAD system are designed to improve the accuracy of lung nodule recognition and reduce the missed detection of nodules. The main contributions are as follows:

1. The classical deep learning network (VGG16) is proposed to be used for deep features extraction of nodule ROI in Lung CAD, and the deep features are combined with handcrafted features as the final feature vector to take into account more comprehensive feature information of nodules. On the one hand, it overcomes the shortcomings of the single handcrafted feature and cannot fully reflect the internal features of the lesions, and on the other hand, it complements the lack of interpretability of the deep features.
2. An improved MKL-SVM algorithm is proposed with a linear convex combination of the polynomial kernel with high generalization ability and the sigmoid kernel function with high learning ability to avoid overfitting during the model training process and increase the model generalization ability.
3. In order to overcome the problems of long training time and complex search process of the grid search method, the swarm intelligence strategy is further introduced for parameter optimization to shorten the training time of the model. At the same time, in view of the problem that the locally optimized PSO algorithm is prone to premature phenomenon and lack of particle diversity, this paper adopts a hybrid swarm intelligent optimization strategy, through the disturbance of speed contraction factor and roulette strategy, in the proposed MKL-SVM algorithm and introduces a global optimization algorithm based on SA and PSO to help particles jump out of the local optimum and better seek the global optimum solution, so as to improve the accuracy of the model to identify lung nodules and reduce the missed detection.

The rest of the paper is organized as follows: Section 2 introduces the research and application of machine-learning algorithm in medical image processing and the combined application of swarm intelligence optimization in machine learning.  Section 3 elaborates the method of cascade feature and the MKL-SVM algorithm of hybrid swarm intelligence optimization in this paper.  Section 4 presents the experimental dataset, parameter settings, experimental results, and analysis. The conclusion of this article and future work are summarized in Section 5.

## 2. Related Works

### 2.1. Lung Nodule Feature Extraction Algorithm

#### 2.1.1. Handcrafted Feature

The handcrafted features in Lung CAD are designed according to doctors' suggestions, and are usually represented by texture features, morphological features, and pixel brightness features. Gonçalves et al. [9] used features based on shape, intensity, and texture to describe the ROI of lung nodules, and finally selected exponential kernel SVM as the classifier, and used the receiver operator characteristic (ROC) curve as a discriminant criterion. The model obtained an area under ROC curve (AUC) value of 0.962 under the Lung Image Database Consortium and Image Database Resource Initiative (LIDC-IDRI) database, which has a better classification result. De Carvalho Filho et al. [10] used Minkowski functional, distance measures, representation of the vector of points measures, and other shape features, which were input into SVM to realize the recognition of benign and malignant lung nodules. The experiment used 1405 nodules (including 394 malignant nodules and 1011 benign nodules) from LIDC-IDRI, and the accuracy and sensitivity reached 93.19% and 92.75%, respectively, which can effectively identify nodules and prevent missed detection.

The traditional method of handcrafted features represents the image through global visual underlying characteristic statistics, but discards image detail. Selecting features manually is a very laborious and heuristic (requiring expertise) method; whether it can be selected well depends heavily on experience and luck, and its adjustment requires a lot of time, while deep learning is about learning features. Although several improved methods based on handcrafted feature extraction can enhance the experimental results, it is difficult to extract all significant features of medical images.

#### 2.1.2. Deep Learning Feature

Deep neural network models have powerful hierarchical feature learning capabilities. CNN can use the original image as input and automatically learn deep features for classification, thus eliminating the need for predefined features [25, 26]. Hua et al. [27] first introduced CNN to the nodule classification in lung CT images, simplified the image analysis process of conventional CAD through deep learning technology, and adopted deep confidence networks and CNN models without the need to calculate handcrafted features. The experimental results showed that the deep learning method can achieve better recognition results. Wang et al. [28] designed a Lung CAD system based on CNN to automatically extract features and detect nodules, and realized the rapid detection of candidate nodules from CT images on LUNA16 dataset. The experiments showed that the maximum sensitivity of the model is 96.8%, which can reduce false positives.

Compared with traditional solutions, deeply structured algorithms can automatically extract feature information and potentially generate valuable features. However, the training of deep models requires large datasets, and there are few large-scale medical image datasets with annotations. The lack of training data is an inevitable problem in medical image processing, which can be solved by fine-tuning CNN models pretrained on large-scale datasets such as natural scene classes through transfer learning [29, 30].

#### 2.1.3. Feature Combination

In recent years, the combination of handcrafted features and deep learning features has been gradually applied to Lung CAD systems. Ho and Gwak [31] combined the four handcrafted features of scale invariant feature transform (SIFT); generalized search trees (GIST), local binary pattern (LBP), and histogram of oriented gradients (HOG) with the deep features extracted by CNN; and combined seven traditional supervised learning classifiers to implement the classification of lung diseases, and the results showed that the combination of features can improve the classification results. Bansal et al. [32] proposed to use ResNet network and morphological technology to extract deep features and handcrafted features, and XGBoost was selected for classification after feature combination, with an experimental accuracy of 88.30%, which was better than the other methods mentioned.

The combination of deep learning features and handcrafted features can take into account the image feature information more comprehensively. On the one hand, handcrafted features have certain interpretability than deep features, and on the other hand, deep features can extract more complete feature information of the image and complement the disadvantage that handcrafted features cannot reflect the internal features of the image.

### 2.2. Lung Nodule Recognition Algorithm

#### 2.2.1. Traditional Machine-Learning Algorithm

Traditional machine-learning methods are widely used in the detection and recognition of pulmonary medical images, and SVM algorithm is the most commonly used [8–11, 31–34]. The SVM algorithm is a traditional machine-learning method based on statistical theory, which can minimize structural errors and maximize geometric edges, and is often used in classification tasks and regression analysis. SVM takes structural risk minimization as the criterion, and takes into account both empirical risk minimization and expected risk minimization [12]. The SVM is implemented in the feature space by mapping the input sample data *x* to a high-dimensional feature space through a nonlinear transformation Φ(*x*) and constructing the optimal classification hyperplane. When constructing the hyperplane in space, using the kernel function *K*(*x*_*i*_, *x*_*j*_) to represent the inner product of Φ(*x*_*i*_) and Φ(*x*_*j*_), the specific form of Φ(*x*) can be known without being explicit, as shown in

(1) $$\begin{array}{c} K\left(x_{i},x_{j}\right)=\Phi \left(x_{i}\right)\cdot \Phi \left(x_{j}\right), \end{array}$$

where *x*_*i*_ is the feature variable of the *i*_th_ sample input.

The kernel function is the core of SVM, and constructing a kernel function suitable for a given problem can improve the performance of the classifier. Li et al. [8] proposed a MKL-SVM algorithm mixed with polynomial kernel and Gaussian kernel to identify lung nodules, which has a higher accuracy of 92.00% compared with the single kernel SVM method. In order to learn from heterogeneous features, Tong et al. [33] proposed a SVM lung nodule classification method based on MKL, in which a polynomial kernel and a radial basis kernel were used for the clinical features of the patient and the deep features of the image, respectively, and the MKL method was able to improve the classification accuracy compared to using the deep neural network alone. Multiple kernel functions can improve the performance of SVM to some extent, and finding the kernel function for a given problem is a major difficulty. The influence of SVM model parameter selection cannot be ignored. The grid search method has a complex training process and a long training time, so the swarm intelligence optimization algorithm stands out.

#### 2.2.2. Parameter Optimization Algorithm

Most machine-learning problems involve an optimization problem, such as Bayesian algorithm based on maximizing the posterior probability, K-means algorithm to minimize the intra-class distance, and SVM algorithm to maximize the classification hyperplane. The objective function or loss function is optimized through optimization methods to train the best model. Optimization algorithms are mainly divided into two categories: gradient algorithms (deterministic algorithms) and gradient-free optimization algorithms (stochastic algorithms) [35]. The gradient algorithm is an iterative approach to find the optimal solution in a determined direction until the algorithm converges to the optimal solution. The gradient algorithm is simple to implement and can find the global solution, which is loved by researchers, but it still has the following disadvantages: convergence slows down when it is close to the optimal solution, it is easy to fall into the local optimum, and it is greatly influenced by the initialization parameters. However, the proposal of stochastic algorithms provides more solutions to problems [36]. The gradient algorithm has a strict moving direction; if the iteration starts from the same initial point, the solutions are the same, which makes the gradient algorithm not flexible enough and does not have diverse solutions. In contrast, the stochastic algorithm searches for solutions in a more flexible manner, which is less influenced by the initialization parameters and is more likely to jump out of the local optimum. Although different solutions may be obtained even with the same initial values, however, they will still converge to the optimal solution within the given range, even though they are slightly different [35].

At present, the more common stochastic algorithms are mainly evolutionary algorithms, swarm intelligence optimization algorithms, etc. Among them, swarm intelligence optimization algorithms are widely used and perform more prominently in solving modern nonlinear numerical global optimization problems. The swarm intelligence optimization algorithm is derived from the idea of natural evolution and follows the following principles: proximity principle, quality principle, diversity response principle, stability principle, and adaptability principle [37, 38]. Swarm intelligence optimization is mainly designed based on the social behavior mechanism of a certain biological group. Each member of the group changes the direction of search by continuously accumulating experience, randomly generating, evolving and updating a large number of possible solutions, until the stopping criterion is reached, then the search stops. The swarm intelligence optimization strategy has the characteristics of fast solving speed, high accuracy, wide application range, and strong stability, and it is widely used in the parameter optimization of machine-learning algorithms [39, 40]. Typical swarm intelligence optimization includes PSO [41], Ant Colony Optimization (ACO) [42], Artificial Bee Colony (ABC) [43], etc. In recent years, new swarm intelligence optimization algorithms such as Grey Wolf Optimizer (GWO), Whale Optimization Algorithm (WOA), and Grasshopper Optimization Algorithm (GOA) have also emerged and excelled in solving a variety of optimization problems such as quadratic planning and convex planning [44–49].

PSO has the advantages of simple operation, faster convergence, and fewer setup parameters, and is a widely used swarm intelligence optimization algorithm [50, 51]. Suppose a population *X*=(*X*_1_, *X*_2_,…, *X*_*n*_) of *n* particles in a *D*-dimensional search space, the position of the *i*_th_ particle in the *D*-dimensional search space is *X*_*i*_=(*x*_*i*1_, *x*_*i*2_,…,*x*_*iD*_)^*T*^, which also represents a potential solution of the problem. The adaptation value corresponding to each particle *X*_*i*_ can be obtained according to the objective function. Suppose the velocity of the *i*_th_ particle is *V*_*i*_=(*V*_*i*1_, *V*_*i*2_,…,*V*_*iD*_)^*T*^, its individual extremum is *P*_*i*_=(*P*_*i*1_, *P*_*i*2_,…,*P*_*iD*_)^*T*^, and the population extremum is *P*_*g*_=(*P*_*g*1_, *P*_*g*2_,…,*P*_*gD*_)^*T*^. During each iteration, the particle updates its velocity and position by individual and population extremes, and the updated expressions are

(2) $$\begin{array}{c} V_{id}^{k+1}=\omega V_{id}^{k}+c_{1}r_{1}\left(P_{id}^{k}-X_{id}^{k}\right)+c_{2}r_{2}\left(P_{gd}^{k}-X_{id}^{k}\right),\\ X_{id}^{k+1}=X_{id}^{k}+V_{id}^{k+1}, \end{array}$$

where *k* is the number of current iterations; *ω* is the inertia weight; *d*=1,2,…, *D*; *V*_*id*_ is the velocity of the *i*_th_ particle in the *d*_th_ dimension; *c*_1_ and *c*_2_ are acceleration factors (nonnegative constants), and *r*_1_ and *r*_2_ are random numbers distributed in the interval [0, 1]. To prevent the blind search of particles, their velocity and position are usually restricted to the range of [−*V*_max_, *V*_max_] and [−*X*_max_, *X*_max_], respectively.

Although the PSO algorithm can get the optimal solution faster, with the increase of the number of iterations, the population diversity will decrease, which is easy to cause precocity, thus falling into the local optimal. The No Free Lunch theorem shows that the average performance of any two optimization algorithms is equal for any optimization problem, and no optimization algorithm performs well in terms of computational efficiency, generality, and global search capability [52]. Therefore, it is a great strategy to solve parameter optimization by combining multiple optimization ideas. Bi and Qiu [53] combined Genetic Algorithm (GA) and SA algorithm to propose an effective global optimization algorithm, and experiments showed that the convergence speed of the algorithm was improved. Mafarja and Mirjalili [54] proposed two hybrid schemes based on SA and WOA for the feature selection problem, one is to embed SA into WOA to enhance the search ability of the population, and the other is to use SA algorithm to further search for the best solution after the solution of WOA algorithm, and the experiments verified that the hybrid approach can improve the classification accuracy.

To improve the overall performance of the PSO algorithm, the researchers have also made improvements in terms of parameter settings, convergence, and combination with other algorithms. For the problem of high-dimensional feature selection, to improve the search speed of the particles, Song et al. [55] combined the feature clustering method to reduce the search space of the PSO algorithm and improved the overall performance through correlation guidance and adaptive disturbance. To improve the robustness of the algorithm, Koessler and Almomani [56] proposed a hybrid optimization algorithm of pattern search and PSO, and the experimental results showed that the hybrid optimization strategy was successful in improving the accuracy and robustness. Tharwat and Hassanien [57] used the quantum-behaved particle swarm optimization based on statistical methods to find the parameters of SVM, by introducing Monte Carlo Methods and the idea of averaging into the standard PSO to increase the randomness of particle positions, which reduces the number of parameters and can reduce the rate of classification errors. Choudhary et al. [58] used the genetic mutation operator in the GA algorithm combined with the PSO algorithm to avoid the phenomenon of premature convergence of particles, and the experimental results showed that the hybrid algorithm performed well in metrics such as optimal solution, mean value, and computation time.

Therefore, the design of appropriate swarm intelligence optimization strategies for different problems can combine the advantages of multiple algorithms to a greater extent, making the model solution faster and more accurate.

#### 2.2.3. Deep Learning Algorithm

Deep learning has made significant contributions to the detection and recognition algorithms for Lung CAD systems in recent years, with CNN being the most widely used [59]. Qin et al. [60] developed a system for automatic detection of pulmonary nodules in CT images using 3D U-Net, 3D DenseNet, and region proposal network (RPN). On the LUNA16 dataset, the sensitivity of the multitask residual learning and hard negative mining method can reach 96.7%, which was better than other methods. Liu et al. [61] constructed a new multiscale multiview model based on CNN for the lung nodule classification problem, with an overall accuracy of 92.1% and 90.3% in the LIDC-IDRI dataset and ELCAP dataset, respectively. Masood et al. [62] proposed a multidimensional region-based fully convolutional network for lung nodule detection and used the LIDC-IDRI dataset to verify the validity of the model, and the experimental results showed that the sensitivity and classification accuracy of the proposed model reached 98.1% and 97.91%, respectively. Deep learning plays an important role in the field of medical images, but the problems of unbalanced samples of medical image datasets, lack of generality and interpretability of network architectures, and high computational cost still remain to be explored.

#### 2.2.4. Combination of Deep Features and Traditional Machine-Learning Methods

In recent years, there has been much interest in combining deep learning with traditional machine learning. Ginneken et al. [17] used a pretrained CNN model for ROI feature extraction combined with linear SVM for classification, using 865 scans of CT from the LIDC-IDRI dataset for their experiments. CNN performed well but not as well as traditional CAD systems for lung nodule detection, and when the two methods were used in combination, significantly better results were obtained than either method alone. Da Nóbrega et al. [18] for lung nodule malignancy classification problem, based on the transfer learning method, proposed feature extraction through networks such as VGG16, VGG19, and ResNet, and then combined with traditional machine-learning methods such as multilayer perceptron, SVM, and RF. The results showed that the combination of the deep feature extractor based on ResNet and traditional SVM had an AUC of 93.1%, which has better classification performance. Polat and Danaei Mehr [19] proposed a new 3D-CNN model based on AlexNet and GoogleNet networks for feature extraction, and used SVM as a classifier for lung nodule classification, and the results showed that deep learning combined with SVM classifier can improve the performance of the architecture.

In summary, combining deep features with handcrafted features can obtain richer feature information. In addition, combining deep learning methods with traditional machine-learning methods can build models with better results and improve classification performance.

## 3. Key Algorithms of the Proposed Lung CAD System

In this paper, we investigate the key algorithms of the Lung CAD system, mainly including lung nodule ROI feature extraction and recognition. In order to obtain more comprehensive ROI feature information of lung nodules, this paper quantifies the doctors' advice into 13-dimensional handcrafted features, and extracts deep features from the VGG16 pretraining model through transfer learning, and combines handcrafted features with deep features. In the nodule recognition algorithm, an improved MKL-SVM algorithm is adopted, and the kernel function adopts the form of a linear convex combination of polynomial kernel and sigmoid kernel to improve the classification performance of SVM, taking into account the generalization ability and learning ability. To accelerate model training, the proposed MKL-SVM algorithm is further improved by combining the hybrid swarm intelligence optimization strategy, aiming at the problem that PSO algorithm is easy to fall into the local optimal solution. In other words, the hybrid strategy of SA and PSO is used to optimize the parameters of the improved MKL-SVM model, so as to quickly find the optimal parameter set.

### 3.1. Feature Extraction and Feature Combination

#### 3.1.1. Feature Extraction

The feature extraction in this paper is divided into two parts: handcrafted feature extraction and deep feature extraction. Among them, the handcrafted feature extracts the 13-dimensional features of the lung nodule candidate ROI according to the doctors' suggestions, including 7-dimensional morphological features, 2-dimensional grayscale features, and 4-dimensional texture features. The details are the same as in [8].

In the deep feature extraction part, due to the difficulty of acquiring images of lung lesions with annotations, the VGG16 model pretrained on the large public dataset (ImageNet) was selected as the deep feature extractor by the transfer learning method, in order to achieve better learning results.

The VGG16 model replaces the large convolution kernel with a small convolution kernel and multiple convolution layers to reduce the number of parameters of the network, and has better recognition accuracy [5]. The lower layer of CNN is composed of alternating convolutional and max-pooling layers, while the higher layer is a fully connected layer. The feature semantic information of the lower layer is less, and the semantic information of the higher layer is more abundant. The core of CNN feature extraction is the convolutional layer and the pooling layer. The extracted feature map is used as the input of the fully connected layer, which contains the richest semantic information of lung nodules and can describe the features more comprehensively. Therefore, in this paper, the weights before the first fully connected layer of VGG16 will be pretrained and transferred to the target network to extract the deep features. Deep convolutional neural networks have deep abstract information but contain a large number of irrelevant and redundant features, which are prone to the dimensional disaster problem. Feature dimensionality reduction can reduce the complexity of computation while eliminating the noise contained in irrelevant features.

Commonly used feature dimensionality reduction methods include linear discriminant analysis (LDA) and principal component analysis (PCA), two classical algorithms. The LDA method is used to perform a new projection on the eigenvalues. After the projection, the distance of data points of different properties is larger, and the distance of data points of the same property is more compact. PCA maps high-dimensional features to low-dimensional space from the perspective of covariance, and expects the variance of the data to reach the maximum in the projected dimension [63]. In order to be able to find the key subset of the original features and reduce unnecessary feature computation and resource consumption, the PCA method is chosen for feature dimensionality reduction in this paper.

#### 3.1.2. Feature Combination

The specific steps of the feature combination algorithm are as follows:

1. Based on the transfer learning method, the pretrained VGG16 model is used as the feature extractor of the target network, and a total of 25088 dimensional features are extracted.
2. According to the cumulative variance contribution rate and the structure of the VGG16 model, while reducing the computational complexity, the extracted deep features are reduced to 98 dimensions by PCA method.
3. Cascading of handcrafted features and deep features to form combined features.
4. Finally, the combined features are input to the MKL-SVM proposed in this paper to realize the recognition of lung nodules.

### 3.2. Lung Nodule Recognition Algorithm

#### 3.2.1. MKL-SVM Algorithm

The selection of the kernel function is the key to SVM, and according to Mercer's theorem, the kernel function has various forms [12]. The kernel functions are divided into linear and nonlinear kernels, where the linear kernel is expressed as

(3) $$\begin{array}{c} K\left(x,x^{\prime }\right)=\left(x,x^{\prime }\right). \end{array}$$

Commonly used nonlinear kernels include polynomial kernel function, radial basis function (RBF), and sigmoid kernel functions, denoted by *K*_poly_, *K*_rbf_, and *K*_sigmoid_, respectively.

(4) $$\begin{array}{c} K_{\text{poly}}\left(x,x^{\prime }\right)={\left(\left(x,x^{\prime }\right)+1\right)}^{d},\\ K_{\text{rbf}}\left(x,x^{\prime }\right)=\exp\left(-\frac{{\left\|x-x^{\prime }\right\|}^{2}}{2g^{2}}\right),\\ K_{\text{sigmoid}}\left(x,x^{\prime }\right)=\tanh\left(a\left(x,x^{\prime }\right)+r\right), \end{array}$$

where *d* represents the degree of the polynomial kernel and takes a positive integer greater than 1; *g* represents the RBF kernel width; and *a* and *r* represent the regulation parameter and displacement parameter of the sigmoid kernel, respectively.

It has been shown that each kernel function has its own advantages and also limitations [64]. The polynomial kernel is a global kernel function that acts not only on points close to the sample center but also on points farther away from the center of the sample, and the generalization ability increases as the degree *d* increases. The RBF kernel is a local kernel function that has a large effect on points near the sample centroid, and this effect diminishes as the distance increases. The RBF kernel has a high learning ability but is overly influenced by parameters and prone to overfitting. The sigmoid kernel has strong nonlinear fitting ability and is also a global kernel function. The SVM using the sigmoid kernel is equivalent to a two-layer perceptron network. The structural risk minimization property of SVM can overcome the problem of local optimal solutions in neural networks, and the result is a global optimal solution, which ensures good generalization to unknown samples and can avoid overfitting.

In addition to the several kernel functions introduced above, kernel functions can be constructed according to the actual needs of the problem. It has been proved that the weighted convex combination form of the kernel function satisfies Mercer's theorem, and is still a kernel function, which can be used in the SVM model [8], as shown in

(5) $$\begin{array}{c} K_{\text{mix}}\left(x,x^{\prime }\right)=\overset{N}{\underset{p=1}{\sum }}\alpha_{p}K_{p}\left(x,x^{\prime }\right),\\ \overset{N}{\underset{p=1}{\sum }}\alpha_{p}=1,\;0<\alpha_{p}<1,p=1,\ldots ,N, \end{array}$$

where *α*_*p*_ is the weight of the *p*_th_ basic kernel function in the multiple kernel function and *K*_*p*_(*x*, *x*′) is the *p*_th_ basic kernel function used. A total of *N* basic kernel functions are used in the multiple kernel function and the sum of their weights is 1.

This paper proposed an improved MKL-SVM algorithm. The kernel function used a polynomial kernel with strong generalization ability and a sigmoid kernel function with strong learning ability for linear convex combination, so as to avoid overfitting in the model training process and increase the model generalization ability.

The specific composition of the multiple kernel function is shown in

(6) $$\begin{array}{c} K\left(x,x^{\prime }\right)=\lambda K_{\text{poly}}\left(x,x^{\prime }\right)+\left(1-\lambda \right)K_{\text{sigmoid}}\left(x,x^{\prime }\right), \end{array}$$

where *λ* is free to adjust the weights of different kernels in the multiple kernel function in the range of (0, 1).

#### 3.2.2. Improved MKL-SVM Algorithm for Hybrid Swarm Intelligent Optimization Strategy

Swarm intelligent optimization is a heuristic algorithm, which mainly simulates the life behaviors of various creatures in nature, such as insects, shoal of fish, birds. They forage for food in a cooperative way, and each organism in the group constantly updates the search direction through accumulated experience [65].

PSO algorithm is a typical swarm intelligence optimization algorithm. The PSO algorithm is simple and easy to implement, with fewer setup parameters, and has been widely used, but it also has the following disadvantages. First, the algorithm is prone to precocity. If there are deviations and unreasonable choices in the design implementation and parameter settings of the algorithm, it will lead to the rapid loss of biodiversity of particles in the search process, making the algorithm prone to local optimal solutions. Second, the convergence rate of the algorithm is slow, which is because the algorithm uses individual extremes and global extremes to update the particle state. Third, the convergence accuracy of the algorithm is not high, mainly because the search step of the algorithm is too large and the local search ability is weak.

SA algorithm is a classic global optimization algorithm, which was first applied in 1983 by Kirkpatrick et al. to combinatorial optimization problems [66]. The physical annealing process of SA consists of the following three parts: the heating process, the isothermal process, and the cooling process. The heating process corresponds to the set initial temperature of the algorithm, the isothermal process corresponds to the Metropolis sampling process of the algorithm, and the cooling process corresponds to the decrease of the control parameters. The Metropolis criterion is the key for the SA algorithm to converge to the global optimal solution, which is to accept the deteriorating solution with a certain probability, so that the algorithm can jump out of the trap of local optimality. Given the initial value of the control parameter in advance, the algorithm randomly selects the current solution from the feasible solutions and follows the iterative process of “generating a new solution ⟶ judging ⟶ accepting or discarding.” As the temperature parameter decreases, a series of Markov chains are generated and the optimal solution to the problem is sought step by step.

**Figure 1:**
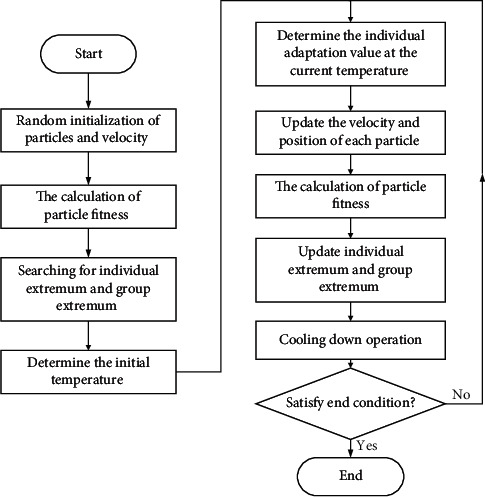
The flow chart of SAPSO algorithm.

To address the problem that the PSO algorithm is prone to the phenomenon of premature maturity and missing particle diversity, this paper adopts a hybrid swarm intelligent optimization strategy, which can effectively avoid particles staying at the local optimum and better seek the global optimal solution. The flow of the proposed SAPSO optimization algorithm for SA and PSO hybrid is shown in Figure 1.

The above steps are specified as follows:

(1) Randomly initialize the position and velocity of each particle in the population.
(2) Calculate the fitness of each particle, and store the current position and fitness value of each particle in *P*(*i*).
(3) Find the individual *P*_best_ with the best fitness value among the current particles, and store the position and fitness value of *P*_best_ in the population extremum *G*_best_.
(4) The initial temperature *T* is generally set empirically, as shown in [67]:
  (7) $$\begin{array}{c} T=\frac{f\left(G_{\text{best}}\right)}{\ln\;5}, \end{array}$$
where *f* is the value of the fitness function.
(5) Determine the fitness value TF(*P*(*i*)) for individuals at the current temperature according to
  (8) $$\begin{array}{c} \text{TF}\left(P\left(i\right)\right)=\frac{e^{-\left(P\left(i\right)-f\left(G_{\text{best}}\right)\right)/T}}{\sum_{i=1}^{N}e^{-\left(P\left(i\right)-f\left(G_{\text{best}}\right)\right)/T}},\\ P\left(i\right)=f\left(X_{i}\right), \end{array}$$
where *f* is the fitness function value and *P*(*i*) is the fitness value of the current particle.
(6) In combination with the roulette strategy, compare the magnitude of the random probability bet with the value of the cumulative probability ComFit(*i*) of an individual particle being selected. Determine the maximum value *G*_plus_ from all *P*(*i*) to replace the global optimal *G*_best_, and then update the velocity and position of the particles according to
  (9) $$\begin{array}{c} \text{ComFit}\left(i\right)=\overset{i}{\underset{m=1}{\sum }}\text{TF}\left(P\left(m\right)\right), \end{array}$$
  (10) $$\begin{array}{c} V_{i}\left(m+1\right)=\varphi \left\{V_{i}\left(m\right)+c_{1}r_{1}\left[P_{\text{best}}\left(i\right)-X_{i}\left(m\right)\right]+c_{2}r_{2}\left[G_{\text{plus}}-X_{i}\left(m\right)\right]\right\},\\ C=c_{1}+c_{2}, \end{array}$$
  (11) $$\begin{array}{c} X_{i}\left(m+1\right)=X_{i}\left(m\right)+V_{i}\left(m+1\right), \end{array}$$
where *m* represents the current particle; *c*_1_ and *c*_2_ are acceleration factors, which affect the trajectory of the particle; *r*_1_ and *r*_2_ are random numbers of [0, 1]; *φ* is the constriction factor, which controls the flying speed of the particles to improve the convergence of the algorithm.
(7) Calculate the new fitness value of each particle, update the individual extreme value *P*_best_ and the group extreme value *G*_best_.
(8) The desuperheating operation is performed according to
  (12) $$\begin{array}{c} T_{i+1}=\lambda T, \end{array}$$
where *λ* is the cooling rate and takes values between [0, 1].
(9) If the maximum number of iterations is satisfied, the search stops; otherwise, go to step (4).

## 4. Experimental Results

**Figure 2:**
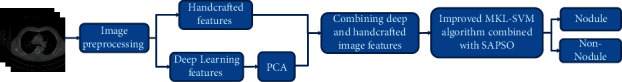
The flow chart of the proposed model.

In this paper, the cascade feature is adopted as the final input feature and the MKL-SVM algorithm is combined with the improved swarm intelligence optimization strategy to identify the nodules. The main purpose is to improve the recognition accuracy and detection rate of the nodules. In order to verify the effectiveness of the proposed key algorithm for CAD system, the algorithm in this paper is applied to lung nodule recognition, and the specific flow of the experiment is shown in Figure 2.

### 4.1. Dataset

The experiment uses a total of two datasets, dataset 1 is the dataset of the cooperative hospital, and dataset 2 is the large public dataset LUNA16 [68]. The experimental dataset 1 comprises 20 sets of CT images acquired from a large specialized hospital in Jilin Province, China, with a total of about 700 images. After the pre-preparation for identification, the specific implementation steps were the same as in reference [8], and a total of 270 candidate ROIs of lung nodules were extracted, including 80 nodules and 190 nonnodules. These data were randomly divided into 170 training samples and 100 test samples, and normalized. In the image preprocessing process, the original image is first grayed out and the isolated type nodule part is individually framed according to the annotation information, and then binarization is performed and the largest 8-connected region is reconstructed to remove the background to obtain the ROI part of the lung nodule.

Experimental dataset 2 is Lung Nodule Analysis 16 (LUNA16), which is a subset of the public LIDC-IDRI database, including 888 groups of low-dose lungs CT images, including 1186 nodules marked by at least 3 radiologists. After the image preprocessing method, a total of 1140 lung nodule ROIs were selected, including 650 nodules and 490 nonnodules. After randomly scattering, 800 of these ROIs were used as the training dataset, and 340 ROIs were used as the test dataset.

In the experiments of this paper, dataset 1 is mainly used for the proposed Lung CAD system key algorithm construction study, while dataset 2 is used to further verify the robustness of the proposed Lung CAD system key algorithm. In this paper, the experiments will be conducted in three aspects, namely, kernel function construction of MKL-SVM, optimization strategy of hybrid swarm intelligence, and feature cascading, respectively. Firstly, for the lung nodule recognition algorithm, in order to select a suitable kernel function and improve the classification performance, experimental dataset 1 is used for algorithm construction experiments. Furthermore, in order to reduce the training time of the model, the swarm intelligence optimization strategy will be used during the experiments, and only 13-dimensional manual features will be used as input. Secondly, based on the selected MKL-SVM recognition algorithm, feature selection experiments were conducted with handcrafted features, deep features, and combined features as input to determine the effectiveness of the feature combination scheme for the Lung CAD system. After the above experiments, the key algorithms of the Lung CAD system in this paper were initially determined. Finally, to further validate the effectiveness of the proposed key algorithm for the Lung CAD system, validation experiments were conducted using dataset 2. In other words, experimental dataset 1 is used for subsequent experiments of SVM algorithms with different kernel functions, experiments of hybrid intelligent optimization strategies, and experiments of feature selection, while dataset 2 is used only for validation experiments of the key algorithms of the proposed Lung CAD system.

### 4.2. Parameter Setting and Evaluation Criteria

#### 4.2.1. Parameter Setting Range

In the optimization stage of the model parameters, the particle population and velocity are initialized first. In the algorithm of this paper, the parameter setting is shown in Table 1.

#### 4.2.2. Evaluation Criteria

In Lung CAD, the accuracy (ACC) index is generally used to measure the overall nodule recognition accuracy, and the sensitivity (SEN) index is used to measure the actual detection rate of lung nodules.

(13) $$\begin{array}{c} \text{ACC}=\frac{\text{TP}+\text{TN}}{\text{TP}+\text{TN}+\text{FP}+\text{FN}},\\ \text{SEN}=\frac{\text{TP}}{\text{TP}+\text{FN}}, \end{array}$$

where TP is the identified true positive nodule; TN is the identified true negative nodule, that is, nonnodule; FP is the false-positive nodule; and FN is the false-negative nodule.

In practice, optimization strategies are often combined with machine-learning algorithms and use ACC or SEN as the fitness function, but it will result in a bias towards the improvement of only one index. In order to take into account both the overall recognition rate ACC and the nodule detection rate SEN to prevent missed detection of nodules, the harmonic mean *F* − *score* function of both is used as the evaluation index in this paper, as shown in

(14) $$\begin{array}{c} F-\text{score}=\frac{2*\text{SEN}*\text{ACC}}{\text{SEN}+\text{ACC}}. \end{array}$$

In the parameter search phase of the model, the *F* − score function under 5-fold cross-validation is used as the fitness function of the proposed algorithm. To ensure the robustness of the experimental results, each subsequent set of experiments is repeated 10 times, and the statistical mean of the 10 experimental results is taken as the final experimental results.

To better evaluate the performance of the model, the following metrics will also be referred to

(15) $$\begin{array}{c} \text{SPE}=\frac{\text{TN}}{\text{TN}+\text{FP}},\\ \text{PRE}=\frac{\text{TP}}{\text{TP}+\text{FP}},\\ \text{MCC}=\frac{\text{TP}*\text{TN}-\text{FP}*\text{FN}}{\sqrt{\left(\text{TP}+\text{FP}\right)\left(\text{TP}+\text{FN}\right)\left(\text{TN}+\text{FP}\right)\left(\text{TN}+\text{FN}\right)}}. \end{array}$$

Among them, Specificity (SPE) represents the correct rate of nonnodule recognition, Precision (PRE) represents the ability of the model to distinguish nonnodules, and Matthews Correlation Coefficient (MCC) represents the correlation coefficient between the predicted classification and the actual classification results. If the SPE and PRE are higher, the FP is lower, and the possibility of judging the nodule as nonnodule is lower, and the false detection is easy to cause psychological stress and additional financial burden to patients, resulting in excessive medical care.

### 4.3. Analysis of Experimental Results

The experiments are divided into two parts: the first part is the experiments for the selection of key algorithms and the second part is the experiments for the validation of the proposed algorithms. Firstly, the selection of key algorithms for lung CAD is performed in dataset 1, which is divided into different kernel function SVM experiments, hybrid intelligent optimization strategy experiments, and feature selection experiments. Secondly, to further determine the robustness of the proposed lung CAD system, validation experiments are performed in public dataset 2.

#### 4.3.1. Experiments of SVM Algorithm with Different Kernel Functions

**Figure 3:**
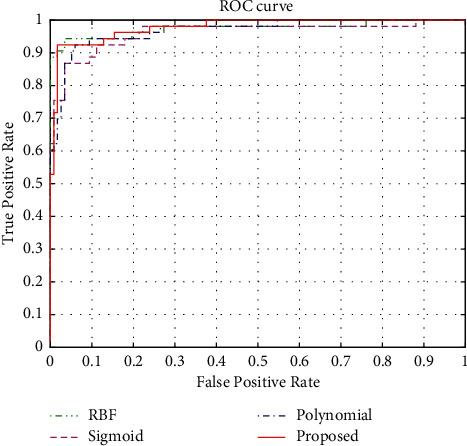
ROC curves of the four kernel functions.

In the comparison experiments of the SVM algorithm using different kernel functions, only the model construction problem is involved and the experimental dataset 1 is used for experimental validation. Several single kernel functions such as RBF kernel, sigmoid kernel, polynomial kernel, and the proposed multicore function shown in equation (6) are selected for the experiments, and the ROC curves during the training phase are shown in Figure 3.  Table 2 shows the AUC for each of the four kernel functions, and the larger the AUC value, the better the classifier effect.

**Figure 4:**
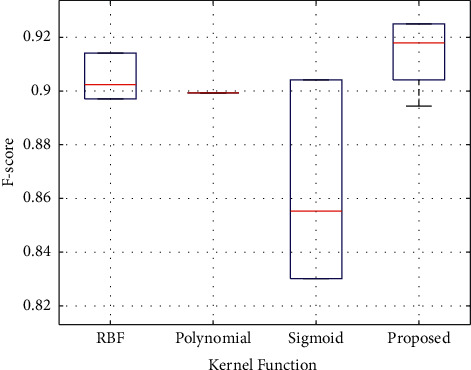
Box plots of the kernel functions in the test stage.

In the kernel function selection problem, as shown in Figure 3, the upper left vertex of the ROC curve of the proposed algorithm is closer to the (0, 1) point, and the AUC value can reach 0.9779, which has a better recognition effect. In the testing stage, the box plots of the four kernel functions are shown in Figure 4.

The box plot of the proposed algorithm is located at the uppermost end and has the largest maximum and median values. The mean value of the *F* − score in this paper is able to reach 0.9141 and the maximum value is 0.9250, which are optimal compared to several single kernel functions. When *F* − score takes the maximum value, the corresponding ACC and SEN reach 89.00% and 96.30%, respectively, and the missed detection rate is low. In a comprehensive comparison, the experimental results of the multiple kernel function are better, so the subsequent recognition algorithms in this paper all adopt the MKL-SVM algorithm with a mixture of polynomial kernel and sigmoid kernel.

#### 4.3.2. Hybrid Intelligent Optimization Strategy Experiment

**Figure 5:**
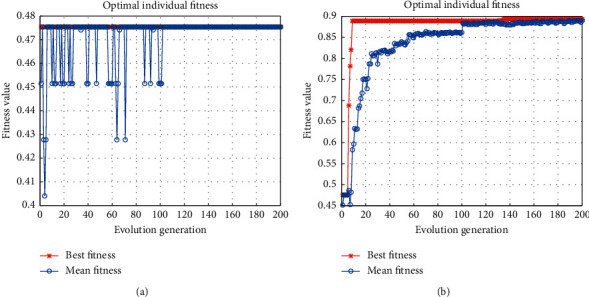
The fitness curve of sigmoid kernel. (a) PSO. (b) SAPSO.

To verify the effectiveness of the proposed hybrid swarm intelligence strategy, the experimental results of different optimization strategies are compared in this section. During the experiments in Section 4.3.1, when only the PSO algorithm was used for parameter search, it was found that the use of the sigmoid kernel was very easy to fall into the local optimal solution in the training phase, as shown in Figure 5(a), and the value of the optimal fitness function obtained was only 0.4753; after using the SAPSO algorithm with hybrid swarm intelligence optimization, it was able to quickly jump out of the local optimal solution trap, as shown in Figure 5(b), and the optimal fitness function value reaches 0.8949. Therefore, in the experiment of 4.3.1, the SAPSO optimization strategy is used.

**Figure 6:**
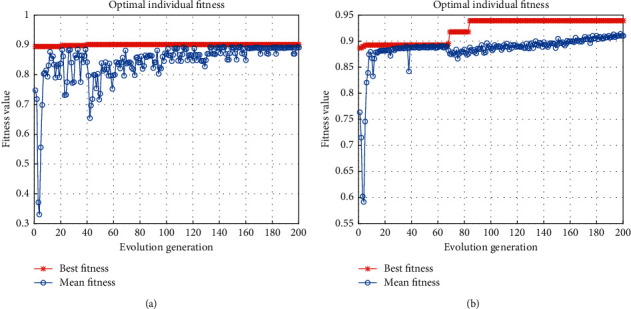
The fitness curve of the proposed recognition algorithm. (a) PSO. (b) SAPSO.

After selecting the multiple kernel functions, the PSO algorithm and the SAPSO algorithm in this paper are experimented with the degree of the multiple kernel functions *d*=2 and *d*=3, respectively. In the training phase, the fitness curve of the algorithm in this paper is shown in Figure 6. The optimal fitness value of 0.9004 is obtained using the PSO algorithm, while the optimal fitness value of 0.9393 is obtained using the SAPSO algorithm, which enables the particles to jump out of the local optimal solution and thus obtain a more optimal value.

Table 3 shows the experimental results obtained in the testing phase for the PSO algorithm as well as the SAPSO algorithm, respectively.

In Table 3, *F*_mean_ is the mean value of *F* − score of 10 experimental results, *F*_max_ is the maximum value, *F*_median_ is the median value, *F*_min_ is the minimum value, ACC_mean_ and ACC_max_ represent the mean and maximum values of ACC of 10 experimental results, respectively, and SEN is the same. SPE, PRE, and MCC are the mean of the results of 10 experiments.

As can be seen from Table 3, it is easier to find the current optimal solution by choosing the SAPSO strategy in the case of *d*=2. The *F* − score related indicators are all optimal, indicating that the SAPSO algorithm is able to balance both ACC and SEN, taking into account the actual detection rate of nodules while ensuring the overall accuracy. Comparing the values of ACC and SEN, the ACC_mean_ and SEN_mean_ of SAPSO algorithm are higher than the PSO algorithm by 0.60% and 2.22%, respectively. Meanwhile, SPE, PRE, and MCC are among the maximum values, which can reduce FP and FN to a certain extent. In the case of *d*=3, the SEN of SAPSO was essentially the same as the PSO algorithm, but ACC_mean_, SPE, PRE, and MCC all improved, indicating that the SAPSO algorithm improved the overall performance of the classifier. Compared to *d*=2, the SEN_max_ of the SAPSO algorithm can reach 100%, but its corresponding ACC is only 77%, which is not satisfactory overall.

In summary, in the subsequent recognition algorithm, the MKL-SVM algorithm based on SAPSO at *d*=2 is used as the final recognition algorithm.

#### 4.3.3. Feature Selection Experiments

Furthermore, 13-dimensional handcrafted features, 98-dimensional deep features, and two feature cascades are used as inputs to the recognition algorithm to determine the final features, respectively.

As shown in Table 4, the *F*_mean_ of the proposed cascade feature is 0.9394, which is better than the single feature. Compared with using handcrafted features alone, *F*_max_ and *F*_min_ improved by 2.81% and 2.14%, and ACC_mean_ and SEN_mean_ improved by 1.4% and 3.71%, respectively. Compared with using deep features alone, *F*_max_ and *F*_min_ improved by 10.76% and 8.23%, and ACC_mean_ and SEN_mean_ improved by 1.3% and 17.72%, respectively. It is worth noting that when only deep features are used, SPE and PRE values are both higher, indicating that FP is less and the possibility of misdiagnosis of nodules as nonnodules is less, but the SEN is low, indicating that there are more FNs and more possibility of missed detection. It is possible that the classification hyperplane is skewed due to the imbalance of the positive and negative samples. However, the comprehensive indexes *F*_mean_ and MCC using cascading features are better, indicating that the proposed algorithm can provide valuable feature information to a certain extent, improve the overall performance of the classifier, increase the actual detection rate of nodules, and avoid the missed detection of nodules.

**Figure 7:**
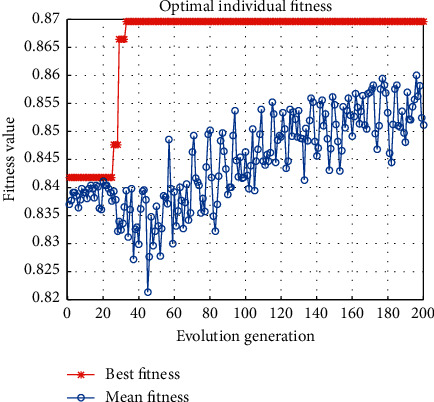
The fitness curve of the algorithm in this paper.

Figure 7 shows the fitness curve of the algorithm in this paper using the combined features. The mean value of the fitness function is 0.8696 for the 10 experiments in the training phase, but the *F*_mean_ value in the testing phase is 0.9394, so it is clear that the generalization ability has improved.

Therefore, cascade features are superior to single handcrafted features or deep features, and the feature selection algorithm in this paper adopts cascade features.

In summary, for the feature selection problem of pulmonary nodules, it was not possible to determine whether a single handcrafted feature or a deep feature was more suitable, but the experimental results of cascading the two types of features were superior; therefore, the model based on cascading the deep features with handcrafted features was selected as the features of the final Lung CAD system. To address the accuracy problem of lung nodule recognition, a linear convex combination of polynomial kernel and sigmoid kernel was used as the kernel function of MKL-SVM, and the *F* − *score* function was used as the final goal, taking into account the double improvement of accuracy and sensitivity. Furthermore, in order to solve the problem that the PSO algorithm was easy to fall into the local optimum in the process of parameter optimization, a swarm intelligence optimization strategy combining SA and PSO was proposed to be applied to the recognition algorithm. Finally, the key algorithms of the proposed Lung CAD system are validated on the LUNA16 dataset.

#### 4.3.4. Key Algorithm Verification Experiments for the Lung CAD System

To further evaluate the effectiveness and generalization of the proposed key algorithms for the Lung CAD system, validation experiments were conducted on the LUNA16 dataset.  Table 5 shows the experimental results of the proposed algorithm and the existing advanced algorithms on LIDC-IDRI and LUNA16 datasets. Since most of the current models are tested using different image data under different conditions, it is impossible to make a detailed and fair comparison with existing solutions. However, by comparing the main statistical metrics of the different methods with the proposal of this paper, excellent results were obtained. As shown in Table 5, the AUC of the proposed algorithm can reach 0.9845, which further validates the generalization ability of the algorithm in this paper. Compared with references [18, 26, 30], the evaluation indexes of the proposed algorithm are all above 90%, and the SPE and PRE are significantly improved, which can effectively avoid the false detection of nodules. Compared with [10, 33, 34], SEN is on par with the existing algorithm, but ACC is improved by about 3%, which has a better overall recognition accuracy. Compared with [25, 62], the performance of the proposed algorithm is slightly lower, but the computational complexity is less than the methods involved in them, which can save the time cost. Subsequently, the training samples can be expanded by adding standard databases to achieve the purpose of improving the classification performance.

## 5. Conclusion

After a series of theoretical studies and experiments on the Lung CAD system, the key algorithms of Lung CAD system based on cascade feature and hybrid swarm intelligence optimization with MKL-SVM were proposed. After experimental validation, firstly, to obtain more comprehensive ROI feature information, handcrafted features and deep features cascade were used. Secondly, to improve the classification performance, a MKL-SVM algorithm in the form of linear convex combination of polynomial kernel function and sigmoid kernel function was proposed. Finally, to solve the problem that the PSO algorithm was easy to lose diversity and fall into the local optimal solution, and to improve the training speed, the SA algorithm and the PSO algorithm were combined to optimize the parameters, and finally applied to the Lung CAD system. In order to further verify the effectiveness of the proposed system, experiments were conducted on the public dataset LUNA16. The results showed the following:

1. The combination of handcrafted features and deep features can preserve feature information as much as possible and improve over using either handcrafted features or deep features alone
2. Compared with the single kernel SVM algorithm, the proposed MKL-SVM algorithm combined with the *F* − score objective function can improve the classification performance of SVM, taking into account both ACC and SEN
3. The SAPSO optimization strategy with a mixture of SA and PSO can make it easier for the particles to seek the global optimal solution and shorten the training time

In summary, the key algorithms of the Lung CAD system proposed in this paper has strong robustness, and can achieve good experimental results on both datasets, which can improve the accuracy of lung nodule recognition and effectively avoid the missed detection of nodules.

In future work, we will explore whether other types of kernel functions can improve the performance of the classifier, and it is also interesting to combine multiple evolutionary algorithms to design optimization schemesand at the same time, to improve the deep learning architecture such as multiple deep features cascade method for feature selection. Meanwhile, it should also be highly focused on the complex calculation problems caused by high dimensional features in the course of more comprehensively retaining the key information of the images. For the problem of lack of datasets with annotation, the data enhancement algorithm is also the next research orientation. In addition, the application of attention models, graph neural networks, embedded strategies, and other technologies to the wise information technology of medicine has yet to be explored.

## Figures and Tables

**Table 1:** Parameter settings of the model.

Number of population particles *n*	20		
Maximum number of population iterations	200		
Per particle dimension *D*	4		
Degree of polynomial kernel *d*	2, 3		
Acceleration factors	*c*_1_ = 1.5, *c*_2_ = 1.7		

Solution of the particle *X*_*i*_=(*x*_*i*1_, *x*_*i*2_, *x*_*i*3_, *x*_*i*4_)^*T*^	Represent the weight share *λ* of the hybrid kernel of the *i*_*th*_ particle, the regularization coefficient *C*, the regulation parameter *a*, and the displacement parameter *r* of the sigmoid kernel, respectively		

Particle settings	Particle	Range of values	Range of velocity
	*λ*	[0, 1]	[-0.6, 0.6]
	*C*	[2^−9^, 2^9^]	[2^−9^*∗*0.6, 2^9^*∗*0.6]
	*a*	[2^−7^, 2^7^]	[2^−7^*∗*0.6, 2^7^*∗*0.6]
	*r*	[−3, 3]	[−3*∗*0.6, 3*∗*0.6]

**Table 2:** AUC values of the four kernel functions.

Kernel function	RBF	Polynomial	Sigmoid	Proposed
AUC	0.9752	0.9686	0.9608	**0.9779**

The bold values represent the maximum values in the row.

**Table 3:** Experimental results of PSO and SAPSO algorithms.

*d*	Algorithm	*F*_mean_	*F*_max_	*F*_median_	*F*_min_	ACC_mean_ (%)	ACC_max_ (%)	SEN_mean_ (%)	SEN_max_ (%)	SPE (%)	PRE (%)	MCC (%)
2	PSO	0.9000	**0.9250**	0.9023	0.8753	89.40	91.00	90.74	96.30	**88.90**	75.47	75.61
2	SAPSO	**0.9141**	**0.9250**	**0.9179**	**0.8944**	**90.00**	**92.00**	**92.96**	96.30	**88.90**	**75.89**	**77.34**
3	PSO	0.8743	0.8764	0.8753	0.8701	86.20	90.00	90.74	**100**	84.93	71.83	71.08
3	SAPSO	0.8829	0.9196	0.8753	0.8701	87.30	91.00	90.00	**100**	86.30	73.23	72.71

The bold values represent the maximum values in the column.

**Table 4:** Recognition results under different feature input patterns.

Algorithm	*F*_mean_	*F*_max_	*F*_median_	*F*_min_	ACC_mean_ (%)	SEN_mean_ (%)	SPE (%)	PRE (%)	MCC (%)
Handcrafted features	0.9141	0.9250	0.9179	0.8944	90.00	92.96	88.90	75.89	77.34
Deep features	0.8415	0.8455	0.8455	0.8323	90.10	78.95	**96.94**	**94.21**	79.10
Proposed features	**0.9394**	**0.9531**	**0.9480**	**0.9158**	**91.40**	**96.67**	89.14	79.71	**81.87**

The bold values represent the maximum values in the column.

**Table 5:** Performance comparison between the proposed algorithm and existing algorithms.

References	Year	Datasets	Methods	ACC (%)	SEN (%)	SPE (%)	PRE (%)	AUC
Filho et al. [10]	2017	LIDC-IDRI (1405 images)	Shape features + GA + SVM	93.19	92.75	93.33	—	0.93
Da Nóbrega et al. [18]	2018	LIDC-IDRI (1536 images)	ResNet50 + SVM	88.41	85.38	—	73.48	0.9313
Zhao et al. [30]	2019	LIDC-IDRI (743 images)	Transfer learning CNNs	85.00	94.00	—	—	0.94
Tong et al. [33]	2020	LIDC-IDRI (1601 images)	3D-CNN + heterogeneous features	91.29	91.01	91.40	—	—
Mastouri et al. [34]	2020	LUNA16 (3186 images)	Bilinear CNN + SVM	91.99	91.85	92.27	—	0.9590
Masood et al. [62]	2020	LIDC-IDRI (892 images)	Enhanced multidimensional region-based fully CNN	97.91	98.10	93.20	—	0.9813
Abid et al. [25]	2021	LIDC-IDRI (2370 images)	Multiview convolutional recurrent neural network	97.10	97.50	97.60	96.70	0.99
Majidpourkhoei et al. [26]	2021	LIDC-IDRI (7072 images)	CNN architectures based on LeNet-5	90.10	84.10	91.70	74.10	—
Proposed	2021	LUNA16 (1140 images)	Cascade features + improved MKL-SVM	95.88	91.97	98.52	97.67	0.9845

## Data Availability

The data of validation experiments used to support the findings of this study are available at https://luna16.grand-challenge.org.
